# Mutation-based structural modification and dynamics study of amyloid beta peptide (1–42): An in*-*silico-based analysis to cognize the mechanism of aggregation

**DOI:** 10.1016/j.gdata.2016.01.003

**Published:** 2016-01-09

**Authors:** Pritam Kumar Panda, Abhaysinha Satish Patil, Priyam Patel, Hetalkumar Panchal

**Affiliations:** a School of Biotechnology and Bioinformatics, D.Y. Patil University, Navi Mumbai, India; b Gujarat Agricultural Biotechnology Institute, Navsari Agricultural University, Athwa Farm, Ghod Dod Road, Surat, Gujarat, India

**Keywords:** Amyloid β peptide, *Alzheimer*'*s disease*, Aggregation, Mutational analysis, NAMD, UCSF Chimera, Discovery Studio Visualizer

## Abstract

Alzheimer's disease is the prevalent cause of premature senility, a progressive mental disorder due to degeneration in brain and deposition of amyloid β peptide (1–42, a misfolded protein) in the form of aggregation that prevails for a prolonged time and obstructs every aspect of life. One of the primary hallmarks of the neuropathological disease is the accretion of amyloid β peptide in the brain that leads to Alzheimer's disease, but the mechanism is still a mystery. Several investigations have shown that mutations at specific positions have a significant impact in stability of the peptide as predicted from aggregation profiles. Here in our study, we have analyzed the mutations by substituting residues at position A22G, E22G, E22K, E22Q, D23N, L34V and molecular dynamics have been performed to check the deviation in stability and conformation of the peptide. The results validated that the mutations at specific positions lead to instability and the proline substitution at E22P and L34P stalled the aggregation of the peptide.

## 1 Introduction

Alzheimer's disease is a neurodegenerative disorder that affects the brain with a continuous loss of memory and inability to respond to stimuli that causes dementia that is most common in old-age people (above 65 years) with late-onset and less common in childhood (before 65 years) with early onset, classified as two stages, respectively [1], [2]. The disorder prevails for a prolonged time and loss of memory worsens, which obstructs with every aspect of daily lives. Personality and behavioral changes affect the social aspects by trouble in interacting with the social environment [5], [6]. Agitation, withdrawal, and appropriate loss of memory and skills are the common symptoms that lead to mortality due to other infections like pneumonia, malnutrition, etc., accounting for less than 5% in early-onset cases [3]. The gene responsible for the disease is APP, which provides instructions for making a protein called amyloid precursor protein found in brain and spinal cord. Though researchers contemplate that it may bind to several other proteins on the surface of the cells or help cells attach to one another, the mechanism and pathogenesis is still a mystery [4]. Several enzymes cut the amyloid precursor protein into two smaller fragments or peptides amyloid precursor protein (APP) and amyloid β peptide, which are released outside the cell. Amyloid β peptide is likely involved in the ability of neurons to change and adapt over time (plasticity) [7], [9], [10].

The mutation in the *APP* gene is responsible for the same accounting for more than 50 different types of mutations [8], which lead to early and late onset of the disease, some of which are discussed later. The most common mutation is V717I (as seen in Fig. 2) that leads to amyloid β aggregation in the brain and forms clumps called amyloid plaques thus releasing β amyloid peptides of 40, 42, 43 residue Aβ peptides. Six mutations in the *APP* gene have been found to cause hereditary cerebral amyloid angiopathy [13], [14], a condition characterized by stroke and a decline in intellectual function (dementia), which begins in mid-adulthood [15], [16]. The Dutch type, the most common of all the types, is caused by the replacement of the amino acid glutamic acid with the amino acid glutamine at position 22 in the protein sequence (Glu22Gln or E22Q). The Italian type and Arctic type are also caused by changes to glutamic acid at position 22. In the Italian type, glutamic acid is replaced with the amino acid lysine (Glu22Lys or E22K), and in the Arctic type, glutamic acid is replaced with the amino acid glycine (Glu22Gly or E22G). The Flemish type is caused by replacement of the amino acid alanine with glycine at position 21 (Ala21Gly or A21G). In the Iowa type, the amino acid aspartic acid is switched with the amino acid asparagine at position 23 (Asp23Asn or D23N). The Piedmont type of hereditary cerebral amyloid angiopathy is caused by the replacement of the amino acid leucine at position 34 with the amino acid valine (Leu34Val or L34V). These mutations lead to aggregation and deposition of amyloid β peptide (1–42) in the brain that are prone to form clusters and accumulate in blood vessels known as plaques thus lead to dementia [9], [10], [11], [12].

In our current research, the reported mutations were subjected to molecular dynamics simulation using NAMD and their deviation (RMSD) was analyzed and plots were generated using chimera. The mutations lead to the formation of β fibril (Alpha helix converted to β sheets) that were predicted using PASTA2.0 and stability of the mutated peptides were analyzed using mutational analysis tools [29] like PolyPhen 2.0 and I-Mutant 3.0 in comparison to wild type amyloid β peptide (1–42).

## 2 Materials and methods

### 2.1 Retrieval of protein structure

**Fig. 1:**
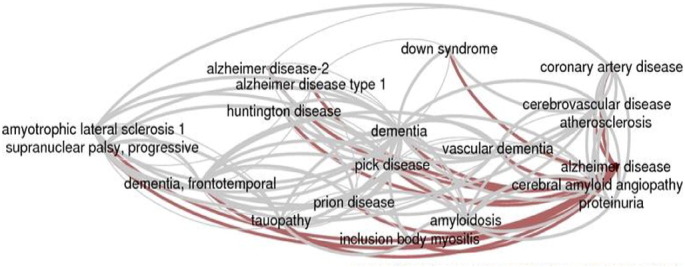
Disorders due to Alzheimer's disease—Weizmann Institute of science [17].

The structural analysis of amyloid β (1–42) has been retrieved from the largest structure repository Protein Data Bank (PDB) having PDB-ID 1IYT [18] (solution structure of the Alzheimer's disease amyloid β peptide (1–42)(Fig. 1). The structure has been subjected to mutational analysis computationally and employed as commencement for molecular dynamics. The sequential perspective to study the amino acid residual substitution, significant mutagenesis, and other functionality was derived from UniprotKB [19] having accession no. P05067 in amyloid β peptide (1–42). Other variants of amyloid β peptides like amyloid β fibrils (PDB-ID-2BEG) have also been retrieved to study the bonding patterns formed due to aggregation of the peptide in Alzheimer's disease.

### 2.2 Mutations and energy calculation

As per the literature review, the amyloid β peptide (1–42) was subsequently mutated at residual positions 21, 22, 23, and 34 using Swiss PDB Viewer [20] to understand the effects, i.e. stability on the peptide during aggregation. The energy force field of the wild type structure, mutated structures was calculated using default parameters. The energy minimization of the wild type as well as the mutated structures was done using steepest descent algorithm with a cutoff of 10 angstrom to check the proportionality of bond angles, improper, torsions, electrostatic bonds. Significant differences have been observed which state the stability of the mutated proteins as compared to the wild type. The stability of the protein is significantly reduced when the energy has been computed using UFF force fields using Swiss PDB viewer as seen in (Table 1). It has been observed that the wild type protein energy when compared to the mutated sites, respectively, is having least energy conformation.

### 2.3 Amyloid prediction server

The aggregation of the amyloid β peptide was experimentally determined, which is the main cause of Alzheimer's disease. For the better understanding of the aggregation process from amino acid sequence of the structure, PASTA [21] (prediction of amyloid structure aggregation) was used that determines the portion of the sequence involved in aggregation with graphical interface, which is more likely to stabilize the cross-β core of fibrillar aggregates as shown in (Fig. 3). The server also provides intrinsic disorder and secondary structure predictions that complement the aggregation data.

## 3 Mutational analysis

### 3.1 I-Mutant 3.0 and PolyPhen 2.0

The Genetics Home Reference from U.S National Library of Medicine gives a detailed functional and structural aspect of the amyloid β precursor protein that elucidates the pathogenesis caused by mutation in specific positions as mentioned above. To have a better insight to the stability of the protein caused by mutation, mutational using I-Mutant 3.0 [22] did analysis where a single-point mutation may lead to several chronic diseases. The study of the effect of a mutation may alter the stability and might lead to pathogenicity as previously reported. PolyPhen-2 (Polymorphism Phenotyping v2) [23] is a tool which predicts possible impact of an amino acid substitution on the structure and function of human proteins that describe the non-synonymous SNP effect. Thus, to analyze the effects of mutation, this tool has been used to encode the damaging effect determined by its sensitivity and specificity score.

### 3.2 Molecular dynamics

Molecular dynamics simulation has emerged as a vital aspect to interpret the intrinsic molecular aspects of several proteins. The state of experimental methodologies has been simplified by simulation approach as it reveals the mechanism involved in atomistic level of the protein. Here in our study, the molecular dynamics simulation approach has been applied to study the stability aspect of our wild and mutated proteins in amyloid β peptide (1–42). For the purpose to identify the effects of mutations, the mutated and wild type proteins were subjected to Scalable Molecular Dynamics NAMD [24] software from Theoretical Biophysics Group, University of Illinois and Beckman Institute, which is a command line program interpreted and altered by Visual Molecular Dynamics [25] (VMD) program.

### 3.3 Types of mutations

The pathogenesis of AD is a hypothesis, which is supported by detailed evidence that argues the accumulation and aggregation of amyloid β peptide. Several missense mutations support the fact that the change in the amino acids leads to onset of AD. The primary references of amyloid precursor protein mutations have been reported earlier since from 1990, soon after the description of pathology of AD by German psychiatrist and near pathologist Alois Alzheimer in 1906 [32]. The common type of mutations are Swedish (1992), Flemish (1992), Dutch (1990), Arctic (2001), Italian (2010), Osaka (2008), Iowa (2001), Austrian (2000), Iranian (2002), French (1999), German (2001), Florida (1997), London (1991), Indiana (1991), and Australian (2000). Experimental evidence by NMR studies identified that a mutations at position E22 and D23 mutations destabilize this turn of the region V24 to K28 (Fig. 6) and thereby promote oligomer formation that results in aggregation [30], [31].

## 4 Results

The amyloid structure aggregation prediction that is a resultant of β sheets from helix stabilized residues in secondary structure conformation was the main viewpoint in this work which was done by using PASTA 2.0. The graphical plots generated thus imply that the residues ranging from 32 to 40 assumed to attain β sheet conformation, i.e. β-amyloid regions. The graph illustrates the parallel aggregation profile in residual range of 32–40. Aggregation free energy profile and strand profile predicted from the server shows that the residues belonging to the residual range 32–40 for formation of aggregates were below the threshold value, which clearly shows the instability index and aggregation of amyloid regions as shown in (Fig. 4a, b, c).

## 5 Dynamics

To study the behavioral and stability aspect of the residues and their conformations due to mutations in protein through MD simulation which depict the intrinsic molecular aspect in atomistic level. The mutated proteins as well as the wild type protein were subjected to VMD for protein structure files generation (PSF), which is a prerequisite for NAMD simulation. The minimization and equilibration of the proteins were done using several parameters. The temperature was set to 310 K with CHARMM force field parameter and integrated time step. i.e. 2 fs/s. The peptide was cantered in a cubic simulation box with a 1 nm distance allowed between the peptide and the edges of the box treated with periodic boundary conditions where it shows the stability of a protein inside a solvent box in which the deviation in the RMSD can be analyzed. LANGEVIN Temperature that is a default parameter in configuration file and PME (for full-system periodic electrostatics) were set to 175 during parameterization. The restart frequency was set to 500 steps for every 1 ps. The minimization and dynamics was done for 10 ps with a frame rate of 1100 that plots a deviation (RMSD) in which the stability of the proteins was analyzed as shown in (Table 2).

### 5.1 Proline substitution

The conformation of amyloid β peptides consists of two α-helical regions that exist at residues 8–25 and 28–38. Other residues apart from the alpha helical regions acquire the coil conformation. Proline acts as a β sheet breaker, which is most common in amyloid β fibrils, that attains a conformation in diseased condition [28]. Insertion of bulky groups or proline within the β sheet regions is effective in inhibition of aggregation as shown in Fig. 5h).

## 6 Discussion

**Fig. 2:**
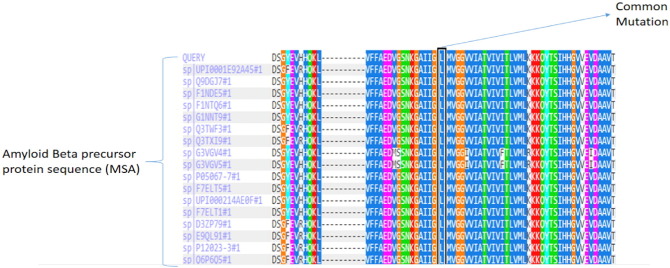
The most common mutation in amyloid β peptide (1–42).

**Fig. 3:**
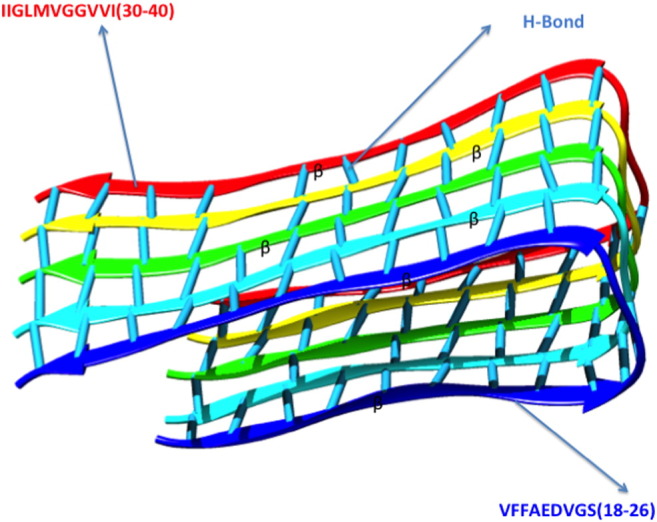
Amyloid fibril formation due to aggregation of β sheets joined by a hinge.

**Fig. 4:**
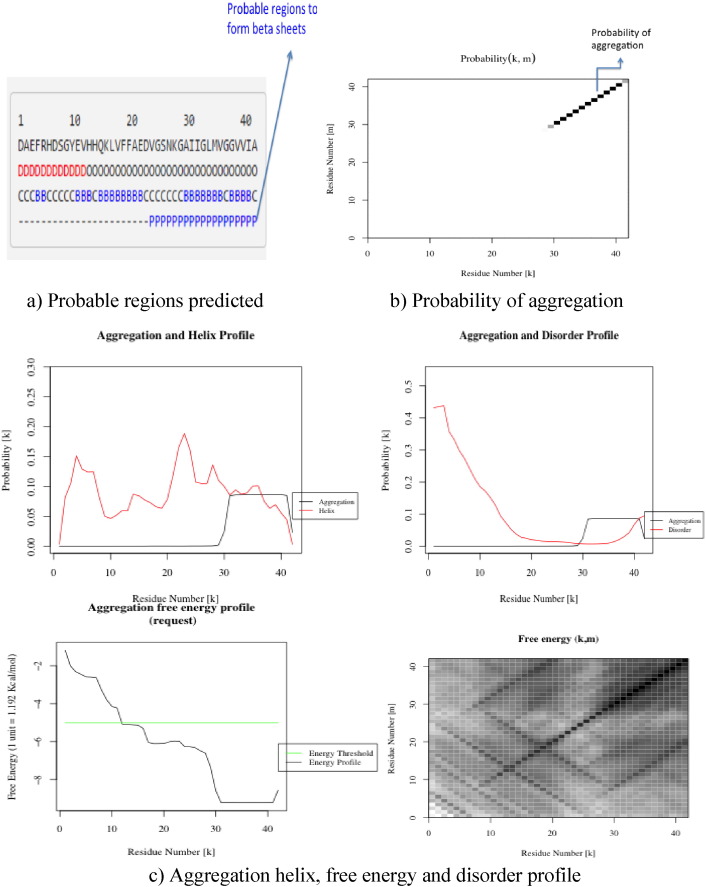
a)Probable regions predicted. b) Probability of aggregation. c)Aggregation helix, free energy, and disorder profile.

**Fig. 5:**
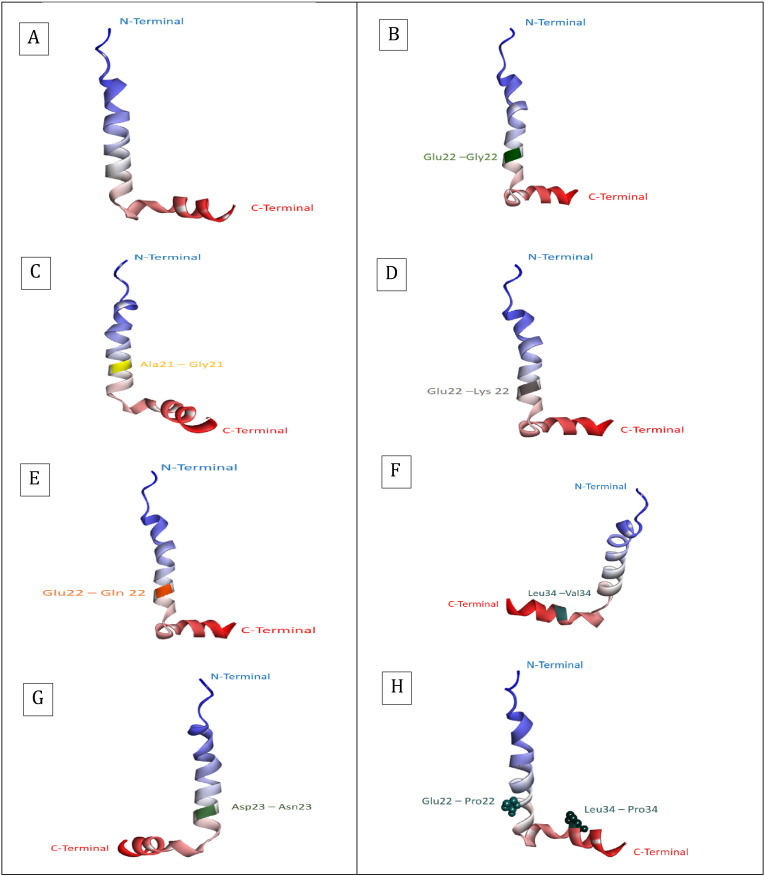
Diagrammatic representation of position specific mutation using Discovery Studio Visualizer 4.1 [26].

**Fig. 6:**
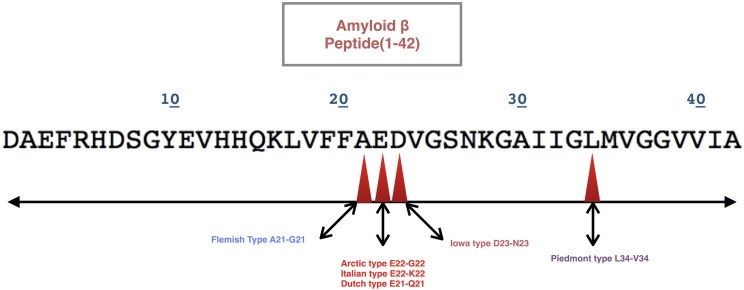
Mutation types at specific positions in amyloid β peptide.

The current work was intended at understanding the effects of mutation and substitution of amyloid β peptide in aggregation. The amyloid β fibril formation is the main cause in Alzheimer's disease that is pointedly related to its pathogenesis. The types of mutations as reported in literature review have been done using Swiss PDB Viewer at specific positions like A22G, E22G, E22K, E22Q, D23N, L34V in amyloid β peptide (1–42) as shown in (Fig. 6) and were subjected to molecular dynamics using NAMD and VMD. Significant changes were observed using UCSF Chimera [27] by the means of RMSD deviation plots and maps, inclining toward the stability effect and chance of formation of amyloid fibrils (β sheets rich conformations) in AD. Despite that the role of amyloid β peptide in formation of β sheet conformations due to aggregation is revealed, the actual mechanism is a mystery. The aggregation profile plots generated by PASTA 2.0 server implies the probability of formation of β sheets at helix positions in the peptide, i.e. (21–42) which clearly indicates that mutational changes affect the helix conformation and subsequently leads to formation of β sheets in due time intervals that is achieved by the RMSD deviation plots generated and simulation approach. The different types of mutations reported were done and their stability were analyzed using I-Mutant 3.0 and PolyPhen2.0 which shows significant decrease in its stability upon mutation. L34V was considered as the common mutation as shown in (Fig. 2), which shows a significant variation as compared to wild type amyloid β peptide (1–42). The substitution of proline that acts as a β sheet breaker were substituted at position E22P and L34P as it provides a link at particular site that further prevents the formation of β sheets (β fibril conformation).

## 7 Conclusion

The pathogenesis of AD is still clandestine, as the mechanism of formation of β sheet conformation as a result of aggregation is not yet understood. The mutation at specific positions leads to instability of peptides as seen in (Table 1, Table 2). Due to substitution of proline, the significant proportionality in reduction of aggregation was observed as it acts as a β sheet breaker. Since the changes observed due to different types of mutations were significant by means of molecular dynamics and the study might lead to understanding of the pathogenesis of AD.

## Figures and Tables

**Table 1:** Mutational analysis.

S. no.	Residue position	Mutated positions	Type of mutation	I-Mutant stability analysis	PolyPhen analysis
1	21	Ala21–Gly21	Flemish type	Decrease	
2	22	Glu22–Gly 22	Arctic type	Decrease	
3	22	Glu22–Lys22	Italian type	Decrease	
4	22	Glu22–Gln22	Dutch type	Decrease	
5	23	Asp23–Asn23	Iowa type	Decrease	
6	34	Leu34–Val34	Piedmont type	Decrease	

**Table 2:** RMSD deviation plot and map of all positional specific mutation in comparison to wild type.

*Structure*	*RMSD value*	*RMSD plot*	*RMSD map*
Wild type	1.394		
Ala21–Gly21	1.248		
Glu22–Gly 22	1.396		
Glu22–Lys22	1.032		
Glu22–Gln22	1.099		
Asp23–Asn23	1.182		
Leu34–Val34	1.532		
Proline substituted (E22P and L34P)	1.266		

## References

[1] Ashford J.W. (2004). APOE genotype effects on Alzheimer's disease onset and epidemiology. J. Mol. Neurosci..

[2] Bird T.D. (Apr 2008). Genetic aspects of Alzheimer's disease. Genet. Med..

[3] Bird T.D. (Mar 3 2005). Genetic factors in Alzheimer's disease. N. Engl. J. Med..

[4] Cummings J.L. (Jul 1 2004). Alzheimer's disease. N. Engl. J. Med..

[5] Ertekin-Taner N. (Aug 2007). Genetics of Alzheimer's disease: a centennial review. Neurol. Clin..

[6] Gene Review: Early-onset familial Alzheimer's disease

[7] Harman D. (May 2006). Alzheimer's disease pathogenesis: role of aging. Ann. N. Y. Acad. Sci..

[8] Janssen J.C., Beck J.A., Campbell T.A., Dickinson A., Fox N.C., Harvey R.J., Houlden H., Rossor M.N., J. C. (Jan 28 2003). Early onset familial Alzheimer's disease: mutation frequency in 31 families. Neurology.

[9] Kamboh M.I. (Jul 2004). Molecular genetics of late-onset Alzheimer's disease. Ann. Hum. Genet..

[10] Li Y., Grupe A. (Dec 2007). Genetics of late-onset Alzheimer's disease: progress and prospect. Pharmacogenomics.

[11] Lott I.T., Head E. (Mar 2005). Alzheimer's disease and Down syndrome: factors in pathogenesis. Neurobiol. Aging.

[12] Lott I.T., Head E. (2001). Down syndrome and Alzheimer's disease: a link between development and aging. Ment. Retard. Dev. Disabil. Res. Rev..

[13] Mattson M.P. (Aug 5 2004). Pathways towards and away from Alzheimer's disease. Nature.

[14] Nussbaum R.L., Ellis C.E. (Apr 3 2003). Alzheimer's disease and Parkinson's disease. N. Engl. J. Med..

[15] Papassotiropoulos A., Fountoulakis M., Dunckley T., Stephan D.A., Reiman E.M. (Apr 2006). Genetics, transcriptomics, and proteomics of Alzheimer's disease. J. Clin. Psychiatry.

[16] Rocchi A., Pellegrini S., Siciliano G., Murri L. (Jun 30 2003). Causative and susceptibility genes for Alzheimer's disease: a review. Brain Res. Bull..

[17] http://www.malacards.org/card/Alzheimer”s_disease? [Search = amyloid + β]

[18] O C., S T., R G., S S., AM D.'.U., PA T., D P. (Nov 2002). Solution structure of the Alzheimer amyloid β-peptide (1–42) in an apolar microenvironment. Similarity with a virus fusion domain. Eur. J. Biochem..

[19] The UniProt Consortium UniProt (2015). A hub for protein information. Nucleic Acids Res..

[20] Guex N., Peitsch M.C. (1997). SWISS-MODEL and the SwissPdbViewer: An environment for comparative protein modeling. Electrophoresis.

[21] Walsh I., Seno F., Tosatto S.C.E., Trovato A. (2014). PASTA 2.0: An improved server for protein aggregation prediction. Nucleic Acids Res..

[22] Capriotti E., Fariselli P., Casadio R. (2005). I-Mutant3.0: predicting stability changes upon mutation from the protein sequence or structure. Oxf. J. Sci. Math Nucleic Acids Res..

[23] Adzhubei I.A., Schmidt S., Peshkin L., Ramensky V.E., Gerasimova A., Bork P., Kondrashov A.S., Sunyaev S.R. (2010). Nat. Methods.

[24] James (2005). Scalable molecular dynamics with NAMD. J. Comput. Chem..

[25] Humphrey W., Dalke A., Schulten K. (1996). VMD – visual molecular dynamics. J. Mol. Graph..

[26] http://accelrys.com/products/collaborative-science/biovia-discovery-studio

[27] Pettersen E.F., Goddard T.D., Huang C.C., Couch G.S., Greenblatt D.M., Meng E.C., Ferrin T.E. (2004). UCSF Chimera – A visualization system for exploratory research and analysis. J. Comput. Chem..

[28] Singh S.K., Singh A., Prakash V.C.S.K. (Sep 30 2014). Structure modeling and dynamics driven mutation and phosphorylation analysis of β -amyloid peptides. Bioinformation.

[29] Panda P.K., Patil S., Patel P. (2015). In-silico stability analysis and phosphorylation induced structural simulation of alpha-synuclein in Parkinson's disease. Research and Reviews. J. Bioinforma..

[30] Grant M.A., Lazo N.D., Lomakin A., Condron M.M., Arai H., Yamin G., Rigby A.C., Teplow D.B. (2007). Familial Alzheimer's disease mutations alter the stability of the amyloid beta-protein monomer folding nucleus. Proc. Natl. Acad. Sci. U. S. A..

[31] Lazo N.D., Grant M.A., Condron M.C., Rigby A.C., Teplow D.B. (2005). On the nucleation of amyloid beta-protein monomer folding. Protein Sci..

[32] S W., D B. (Mar 30 2012). Molecular consequences of amyloid precursor protein and presenilin mutations causing autosomal-dominant Alzheimer's disease. Alzheimers Res. Ther..

